# Genome-wide association analysis of the strength of the MAMP-elicited defense response and resistance to target leaf spot in sorghum

**DOI:** 10.1038/s41598-020-77684-w

**Published:** 2020-11-30

**Authors:** Rozalynne Samira, Jennifer A. Kimball, Luis Fernando Samayoa, James B. Holland, Tiffany M. Jamann, Patrick J. Brown, Gary Stacey, Peter J. Balint-Kurti

**Affiliations:** 1 Department of Entomology and Plant Pathology, North Carolina State University, Raleigh, NC 27695-7613 USA; 2 Department of Agronomy and Plant Genetics, University of Minnesota, 1991 Upper Buford Circle, St. Paul, MN 55108 USA; 3 Department of Crop and Soil Sciences, North Carolina State University, Raleigh, NC 27695-7620 USA; 4 Department of Crop Sciences, University of Illinois, 1102 S. Goodwin Ave, Urbana, IL 61801 USA; 5 Department of Plant Sciences, UC Davis, One Shields Ave, Davis, CA 95616 USA; 6 Divisions of Plant Science and Biochemistry, University of Missouri, Columbia, MO 65211 USA; 7 USDA-ARS Plant Science Research Unit, Raleigh, NC 27695 USA; 8 Fiber and Biopolymer Research Institute (FBRI), Department of Plant and Soil Science, Texas Tech University, Lubbock, TX USA

**Keywords:** Agricultural genetics, Plant immunology

## Abstract

Plants have the capacity to respond to conserved molecular features known as microbe-associated molecular patterns (MAMPs). The goal of this work was to assess variation in the MAMP response in sorghum, to map loci associated with this variation, and to investigate possible connections with variation in quantitative disease resistance. Using an assay that measures the production of reactive oxygen species, we assessed variation in the MAMP response in a sorghum association mapping population known as the sorghum conversion population (SCP). We identified consistent variation for the response to chitin and flg22—an epitope of flagellin. We identified two SNP loci associated with variation in the flg22 response and one with the chitin response. We also assessed resistance to Target Leaf Spot (TLS) disease caused by the necrotrophic fungus *Bipolaris cookei* in the SCP. We identified one strong association on chromosome 5 near a previously characterized disease resistance gene. A moderately significant correlation was observed between stronger flg22 response and lower TLS resistance. Possible reasons for this are discussed.

## Introduction

Sorghum (*Sorghum bicolor*) has a diploid genome of ~ 730 Mb with 10 chromosomes^1,2^. It is a widely-grown cereal crop used as feed or silage for animal consumption, for bio-fuel production and as gluten-free grain for human consumption and is better adapted to grow under high heat and drought conditions than other agriculturally important crops like corn and wheat. These agronomically-important traits make the species an attractive crop for the mass production of grains and bio-fuel under challenging growing conditions.

The warm and humid conditions under which much sorghum is grown support the growth of a wide variety of foliar fungi. Among many diseases, target leaf spot (TLS) caused by the necrotrophic fungus *Bipolaris cookei,* is one of the most economically-important fungal diseases of sorghum in the southeastern US, causing major yield losses^3^. TLS causes distinctive oval or elliptical reddish-purple spots that eventually coalesce during disease progression.

The genetic basis of TLS resistance in sorghum has been the subject of several studies. A major recessive resistance gene *ds1* on chromosome 5 was identified as a loss of function allele of a gene encoding a leucine-rich repeat receptor kinase^4^. Other work identified QTL for three different fungal diseases; target leaf spot, zonate leaf spot and drechslera leaf blight, co-localized on chromosome 6^5^. A third study identified a TLS resistance QTL on chromosome 3, as well as the previously-reported chromosome 6 QTL^6^. A recent study identified novel QTL on chromosomes 3, 4 and 9 as well as a strong QTL on chromosome 5 near the *ds1* locus^7^.

Plants possess cell-surface receptors known as pattern recognition receptors (PRRs) that mediate recognition of highly conserved structural molecules associated with microbes known as microbe-associated molecular patterns (MAMPs). The two best-studied MAMPs are bacterial flagellin, especially its flg22 epitope, and chitin, a component of the fungal cell wall^8,9^. MAMP recognition elicits a basal response at the infection site known as MAMP-triggered immunity (MTI) which often includes phenomena such as callose deposition, changes in membrane ion flux, changes in phytohormone concentrations, induction or repression of plant defense-related genes, and production of reactive oxygen species (ROS) and nitric oxide (NO)^10^. In some cases, a pathogen adapted to a particular host can overcome MTI by producing so-called effector proteins, which are usually introduced into the cytoplasm and may suppress MTI. Effectors are sometimes recognized by cytoplasmic receptors known as R proteins, eliciting a strong response known as effector-triggered immunity (ETI) which is quantitatively stronger though, qualitatively somewhat similar to MTI^11,12^.

Non-host resistance can be defined as: “Resistance shown by an entire plant species against all known genetic variants (or isolates) of a specific parasite or pathogen”^13,14^. It has been hypothesized that MTI is a significant cause of non-host resistance, as most non-adapted pathogens cannot subvert the MTI-based defenses of their non-host plants^15^.

Host resistance can be subdivided into qualitative and quantitative resistance. Qualitative resistance is typically based on the action of a single, large-effect gene, while quantitative resistance is mediated by large numbers of small-effect genes^16,17^. There is some evidence that variation in the strength of MTI may underlie some part of quantitative resistance. Genetic variation in the strength of the MTI response has been documented in a number of plant species including brassicas^18–21^, maize^22^, soybean^23^, and tomato^24^ and in several of these cases, QTL controlling the variation were identified. In particular, the fact that several genes resembling PRRs confer quantitative resistance in various plant species^16^ and that the strength of flg22 perception is negatively correlated with susceptibility to *Pseudomonas syringae* in Arabidopsis^20^ suggest that there may be some connection between variation in the MTI response and quantitative disease resistance. However, the relationship between these traits is not well understood, especially in crop plants. The objectives of this study were to characterize the genetic control of the MAMP response and TLS resistance in a diverse panel of sorghum germplasm and to determine if there was evidence of shared genetic control of these traits. Specifically, we wanted to determine whether a stronger MAMP response was indicative of stronger quantitative resistance.

## Materials and methods

### Plant and pathogen materials

A sorghum association mapping population known as the sorghum conversion population (SCP) was provided by Dr. Pat Brown at the University of Illinois (now at UC Davis). It has been described previously^25^ and is a collection of diverse lines converted to photoperiod-insensitivity and smaller stature to facilitate the growth and development of the plants in US environments^26^. 510 lines from this population were used in this study although due to bad germination and other quality control issues, not all the lines were used in the analysis of all three traits. Ultimately data from 345 lines were used for the analysis of the chitin response, 472 lines for the flg22 response, and 456 for TLS resistance. *B. cookei* strain LSLP18 was obtained from Dr. Burt Bluhm at the University of Arkansas.

### MAMP response measurement

Two different MAMPs were used in this study flg22, (Genscript catalog# RP19986), and chitin (Sigma catalog # C9752). Sorghum plants were grown in inserts laid on flats filled with soil (33% Sunshine Redi-Earth Pro Growing Mix) in the greenhouse. Plants were watered the day before sample collection to avoid extra leaf moisture on the day of collection.

The lines were randomized and, for logistical reasons, were planted in batches of 60 lines. For each line, three ‘pots’ were planted with two seeds per line. Subsequent batches were planted as soon as the previous batch had been processed until the entire population had been assessed. Two experimental runs were conducted for both MAMPs with genotypes re-randomized in each of the two runs.

ROS assays were carried out as previously described^27^. Briefly, for each line, six seeds were planted in 3 different pots. From the resulting seedlings, three were selected based on uniformity. Seedlings that looked unusual or were significantly taller or shorter than the majority were not used. Four leaf discs of 3 mm diameter were excised from the broadest part of the 4^th^ leaf of three different 15-day old sorghum plants. One disc per leaf from two plants and two discs from one plant, with the second disc becoming the water control (see below). The discs were individually floated on 50 µl H_2_0 in a black 96-well plate, sealed with an aluminum seal to avoid exposure to light, and kept at room temperature overnight. The next morning a reaction solution was made using 2 mg/ml chemiluminescent probe L-012 (Wako, catalog # 120-04891), 2 mg/ml horseradish peroxidase (Type VI-A, Sigma-Aldrich, catalog # P6782), and 100 mg/ml Chitin or 2 μM of Flg22. 50 µl of this reaction solution was added to three of the four wells. The fourth well was a mock control, to which the reaction solution excluding the MAMP was added. Four blank wells containing only water were also included in each plate.

After adding the reaction solution, the luminescence was measured using SynergyTM 2 multi-detection microplate reader (BioTek) every 2 min for 1 hr. The plate reader takes luminescence measurements every 2 min during this 1 h. The sum of all 31 readings was calculated to give the value for each well. The estimated value for the MAMP response for each genotype was calculated as (average luminescence value of the three experimental wells—the mock well value) -minus the average blank well value. The blank well values were consistently close to zero.

Leaf discs of *Nicotiana benthamiana*, one high responsive sorghum line (SC0003), and one low responsive sorghum line (PI 6069) were also included as controls in each 96-well plate for quality control purposes.

### *B. cookei* inoculum preparation and inoculation

*B. cookei* inoculum was prepared as described previously^28^. Briefly, sorghum grains were soaked in water for three days, rinsed, scooped into 1L conical flasks and autoclaved for an hour at 15psi and 121 °C. The grains were then inoculated with about 5 ml of macerated mycelia from a fresh culture of *B. cookei* LSLP18 isolates and left for 2 weeks at room temperature, shaking the flasks every 3 days. After 2 weeks, the fungus infested sorghum grains were air-dried and then stored at 4 °C until field inoculation. The same inoculum was used for the entire trial and made fresh every year. For inoculation, 6–10 infested grains were placed into the whorl of 4–5 week old sorghum plants. The spores produced from these fungi initiated infection in the young sorghum plants within a week.

### Seed preparation

Before planting in the field sorghum seed was treated with a fungicide, insecticide, and safener mixture containing ~ 1% Spirato 480 FS fungicide, 4% Sebring 480 FS fungicide, 3% Sorpro 940 ES seed safener. Then the seeds were air-dried for 3 days which provided a thin coating of this mix around the seeds. The safener allowed the use of the herbicide Dual Magnum as a pre-emergence treatment.

### Evaluation of Target Leaf Spot resistance

The SCP was planted at the Central Crops Research Station in Clayton, NC on June 14–15 2017 and June 20, 2018 in a randomized complete block design with two experimental replications in each case. Experiments were planted in 1.8 m single rows with a 0.9 m row width using 10 seeds per plot. Two border rows were planted around the periphery of each experiment to prevent edge effects. The experiments were inoculated on July 20, 2017 and July 20, 2018 at which point the sorghum plants were at growth stage 3. Ratings were taken on a one to nine scales (Fig. S2), where plants showing no signs of disease were scored as a nine and completely dead plants were scored as one (Fig. S2). Two ratings were taken in 2017 and four readings in 2018 starting two weeks after inoculation each year. sAUDPC (standardized area under disease progression curve) was calculated as described previously^29,30^.

### Statistical analyses

All statistical analysis of phenotypic data was performed using SAS V9.4 software. The LSmeans of two replicates for each year were calculated and these were used in turn to calculate the overall LS means. Analysis of variance (ANOVA) and least square (LS)means were calculated using the Proc Mix and Proc GLM procedure in SAS respectively. Correlations were calculated using the CORR procedure of SAS^31^.

### Phenotypic data transformation for association analysis

The phenotypic distribution was right skewed for flg22 and chitin-elicited ROS response traits. From a simple ANOVA we determined that higher predicted score values of these phenotypic traits were moderately associated with higher residuals. Therefore, natural logarithm transformation and root square transformation were performed using the raw scores of chitin and flg22, respectively. After transformation, the phenotypic distribution of each trait was less skewed and the relationship between residual and predicted values was improved. Transformed data were used in the downstream association analysis. Each trial was analyzed separately with SAS mixed model procedure in SAS software version 9.4 (SAS institute. 2019). For the chitin and flg22 response, a best linear unbiased estimator (BLUE) was obtained to estimate each line mean phenotypic value by a mixed model considering inbred lines as fixed effects and replications as random effects. Similarly, for TLS, the data of both years were combined by using a mixed linear model across years considering years and replication within years as random effects and inbred lines as fixed. All the original phenotypic data used for analysis is provided in File S1.

### Genotypic data

All genotypic data used for this study are available upon request from the corresponding author. Genotypic data for the SCP were obtained from Dr. Tiffany Jamann and Dr. Patrick Brown (University of Illinois). The original array consisted of ~ 1.12 million SNPs derived from whole-genome sequencing. We used the genotypes of each set of plants with phenotypic data described above. Each data set was first filtered to exclude SNPs with less than 5% minor allele frequency (MAF) and more than 10% heterozygosity. Linkage disequilibrium-based pruning of genotypic data was performed in software Plink v1.9^32^. After pruning, a set of ~ 58 K SNPs were used to compute the kinship matrix in Tassel 5^33^. Pruned data was also used to perform principal component analysis (PCA) with JMP Genomics 9 (SAS, Institute. 2019). Based on PCA, approximately 20% of the variability was accounted for by the first three principal components. To control for population structure in the association analysis, we excluded 37 inbred lines that explain more than 7% of the variability (Fig. S1). After removing outlier inbred lines, a second filter (< 5% MAF and > 10% heterozygote sites) was performed in each data set. The final sub-set of genotypes used in Genome wide Association Studies (GWAS) contained ~ 755 K SNPs for TLS and flg22 and ~ 750 K SNPs for Chitin. The genotypic datasets used for the analysis of the TLS, flg22 and chitin analyses are available from the corresponding authors.

### Association analysis

Genome-wide association analysis based on a mixed linear model (MLM) was performed in Tassel 5^33^. The MLM model used was y = Xβ + Zu + e where **y** is the vector of phenotypes (BLUEs), **β** is a vector of fixed effects, including the SNP marker, tested, **u** is a vector of random additive effects (inbred lines), **X** and **Z** represent matrices, and e is a vector of random residuals. The variance of random line effects was modeled as Var(u) = **K** ${\sigma }_{a}^{2}$, where **K** is the *n* × *n* matrix of pairwise kinship coefficient and ${\sigma }_{a}^{2}$ is the estimated additive genetic variance. A threshold to declare significance of 1/*m* where *m* is the number of markers tested was used^34,35^.

### Candidate gene selection

Genes within 100 Kb of the highly significant markers were considered candidate genes. Identification and annotation of the candidate genes were performed using the maize BTx623 reference genome v3 available on the Ensembl Plants browser. Functional annotation of the candidate gene was based on EnsemblPlants and Gramene annotation.

## Results and discussion

### Evaluation and mapping of TLS resistance

**Figure 1:**
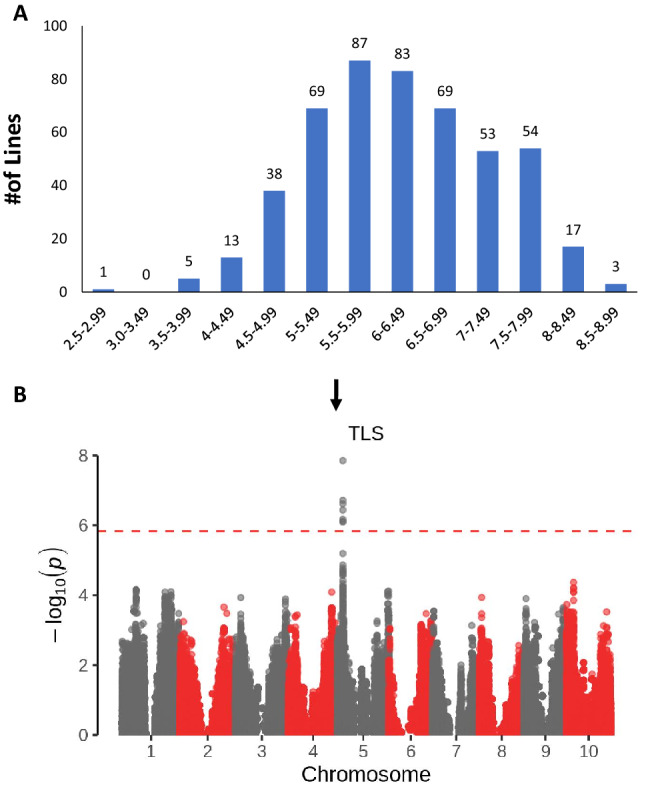
(**A**) Distribution of TLS resistance scores in the SCP. Lines were scored on a 1–9 scale with 9 being immune. The number of lines in each group is indicated at the top of each bar. (**B**) A Manhattan plot of GWAS of TLS resistance in SCP. X-axis indicates position in the genome. Different chromosomes are illustrated as alternating grey and red bands; Y-axis indicates log_10_(p). The Bonferroni multiple comparison test correction significance threshold is indicated. Significant SNPs are indicated by the arrow.

The SCP was assessed in the field for TLS resistance in 2017 and 2018, in randomized complete blocks with two complete replications per year. We observed substantial variation in TLS resistance in the SCP (Fig. 1A). The two replicates in each year were significantly correlated (0.52 and 0.68 in 2017 and 2018 respectively, *p* < 0.0001) and the LSmean scores were significantly correlated between years (0.45, *p* < 0.0001) (Table 1). ANOVA analysis indicated that the genotype effect was significant (Table 2).

**Table 1:** Pearson correlation coefficients among the different phenotypic datasets used in this study.

	TLSR2018^b^	TLS LSmeans^c^	FLG22R1^d^	FLG22R2^e^	FLG22 LSmeans^f^	ChitinR1^g^	ChitinR2^h^	Chitin LSmeans^i^
TLS 2017^a^	0.45***	0.88***	− 0.07	− 0.07	− 0.08	0.05	− 0.02	0.00
TLS 2018		0.79***	− 0.03	− 0.11	− 0.06	− 0.02	− 0.01	− 0.02
TLS LSmeans			− 0.10	− 0.08	− 0.13*	0.01	− 0.02	− 0.02
FLG22R1				0.50***	0.93***	0.07	0.03	0.05
FLG22R2					0.82***	0.20**	0.17*	0.17*
FLG22 LSmeans						0.12*	0.07	0.10
ChitinR1							0.38***	0.81***
ChitinR2								0.89***

***p < 0.0001, **p < 0.001, *p < 0.05.

^a^Target Leaf Spot score from the 2017 field experiment.

^b^Target Leaf Spot score from the 2018 field experiment.

^c^ LSmeans of Target Leaf Spot scores.

^d^1st replication of flg22 response assay.

^e^2nd replication of flg22 response assay.

^f^LSmeans of flg22 response assay.

^g^1st replication of chitin response assay.

^h^2nd replication of chitin response assay.

^i^LSmeans of chitin response assay.

**Table 2:** Analysis of variance of the three phenotypic datasets measured in this experiment.

Traits	Source	DF	Mean Square	F Value
Target leaf spot	Genotype	458	3.169787	81.15*
	Year	1	372.172056	9527.6**
	Rep(Year)	2	2.251893	57.65*
	Genotype*Year	421	1.107162	28.34*
	Genotype*Rep(Year)	772	0.615384	15.75
flg22 response	Genotype	450	383.1881	2.49***
	Replication	1	34.1073	0.22
	Sub sample(Replication)	4	180.7329	1.18
	Line*Replication	446	73.1098	0.48
	Line*Sub sample(Replication)	1786	33.3312	0.22
Chitin response	Genotype	386	12,631.16	17.74**
	Replication	1	1,908,713	2680.35***
	Sub sample(Replication)	4	3941.685	5.54*
	Line*Replication	298	5517.596	7.75*
	Line*Sub sample(Replication)	1368	1636.843	2.3

***p < 0.0001, **p < 0.001, *p < 0.05.

Association analysis using the LSMeans of the 492 lines that were scored identified a single highly significant association on chromosome 5 (Fig. 1B). Table 3 shows the parameters associated with this locus and details predicted genes located 100 Kb either side in the Btx623 genome. One of these genes is *ds1*, a leucine-rich repeat serine/threonine protein kinase gene that was previously reported as a major TLS resistance gene^4^. It seems very likely that this gene underlies the major QTL identified in the SCP.

**Table 3:** List of candidate genes and associated parameters from GWAS of three different traits in SCP.

Trait	SNP	Chr*	P-value	Allele	Effect	Obs^1^	Gene ID	Gene name	Functional annotation	Location
TLS	Chr05pos7332596.1	5	1.41E−08	A/C	0.72909	114/331	SORBI_3005G065100	F-box-like_dom_sf		Chr 5: 7,336,899–7,341,930
							SORBI_3005G065050	Transmembrane helix	Membrane component	Chr 5: 7,307,435–7,307,814
							SORBI_3005G065200	Uncharacterized protein		Chr 5: 7,344,037–7,345,058
							SORBI_3005G065300	SAM-dependent_MTases	Transmembrane transferase	Chr 5: 7,349,434–7,353,139
							SORBI_3005G065000	ds1/ LRR	Ser/Thr protein kinase family	Chr 5: 7,303,703–7,308,391
							SORBI_3005G065400	DUF1618 domain-containing protein		Chr 5: 7,362,167–7,363,717
							SORBI_3005G064900	Cyt_P450	Fe binding transmembrane helix	Chr 5: 7,284,317–7,286,285
							SORBI_3005G064800	Aldehyde dehydrogenase		Chr 5: 7,268,869–7,273,754
flg22	Chr04pos67765644.1	4	4.4E−06	G/T	− 0.3946	439/28	SORBI_3004G348400	Coumaroyltransferase	Transferring acyl group	Chr 4: 67,766,000–67,768,720
							SORBI_3004G348500	Metal binding oxidoreductase		Chr 4: 67,756,085–67,757,761
							SORBI_3004G348700	Znf_RING	Integral component of membrane	Chr 4: 67,758,209–67,760,350
							SORBI_3004G348600	TPR_ domain-containing protein	Protein–protein interaction	Chr 4: 67,759,900–67,763,284
							SORBI_3004G347900	Serine/threonine-protein kinase		Chr 4: 67,717,774–67,722,369
							SORBI_3004G348000	ABC transporter domain-containing	ATP binding ATPase activity	Chr 4: 67,726,322–67,731,341
							SORBI_3004G348200	Protein kinase domain-containing	Protein kinase activity	Chr 4: 67,743,314–67,744,912
	Chr04pos50675144.1	4	5.6E−06	G/T	0.38424	54/407	SORBI_3004G160000	C2H2-type domain-containing protein	nucleic acid binding	Chr 4: 50,673,642–50,677,699
							SORBI_3004G160100	Potassium transporter	K ion trans membrane transport	Chr 4: 50,666,324–50,669,927
Chitin	Chr05pos2510860.1	5	1.3E−06	G/T	− 1.7791	91/233	SORBI_3005G028200	PPR	Sequence specific RBP	Chr 5: 2,515,487–2,517,618
							SORBI_3005G028000	SAUR_fam	Auxin responsive	Chr 5: 2,495,072–2,495,735
							SORBI_3005G028100	Noc2p family	Floral meristem determinacy	Chr 5: 2,498,210–2,504,094
							SORBI_3005G028300	PPR	Sequence specific RBP	Chr 5: 2,530,040–2,533,912
							SORBI_3005G028400	ATPase	Calcium-transporting ATPase	Chr 5: 2,541,308–2,546,634
							SORBI_3005G027840	PPR	Sequence specific RBP	Chr 5: 2,460,971–2,464,621
							SORBI_3005G028600	RING-type domain-containing protein	Ubiquitin protein ligase activity	Chr 5: 2,548,326–2,560,014
							SORBI_3005G028500	NAC domain-containing protein	Transcription regulation	Chr 5: 2,546,682–2,548,092

*Chr = Chromosome, ^1^Obs = Observations. Shows the number of lines genotyped with the two alternate alleles.

### Evaluation and mapping of the MAMP response

**Figure 2:**
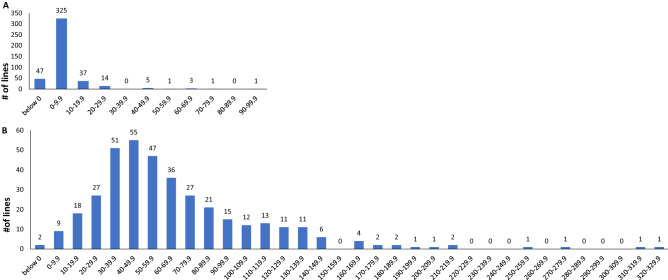
Distribution of phenotypic means of (**A**) flg22- and (**B**) chitin-induced ROS response in the SCP. The number of lines in each group is indicated at the top of each bar.

To assess variation in basal immune response, we measured ROS production in response to flg22 and chitin treatment in the SCP in two full replications. We observed significant variation in response to both MAMPs (Fig. 2). Significant correlations were observed between replicates in both cases (0.5 and 0.38 for flg22 and chitin respectively, *p* < 0.0001, Table 1). ANOVA indicated that genotype effects were highly significant for both traits (Table 1).

The LSmeans of the flg22 and chitin responses were not significantly correlated, though replicate 2 of flg22 response was somewhat correlated with rep1 and rep2 of the chitin response (0.20 and 0.17 respectively, *p* < 0.01 and < 0.05). In previous work, we observed significant correlations in flg22 and chitin responses measured using the same ROS plate assay as well as shared QTL, in a maize recombinant inbred line mapping population^22^. The lack of correlation here is therefore somewhat surprising. It is not clear whether this reflects fundamental differences between the maize and sorghum MAMP responses. Vetter et al^21^ found a negligible correlation in plant growth responses between the bacterial MAMPs EF-Tu and flagellin in Arabidopsis, we are not aware of other published work comparing variation in the responses to two different MAMPs.

**Figure 3:**
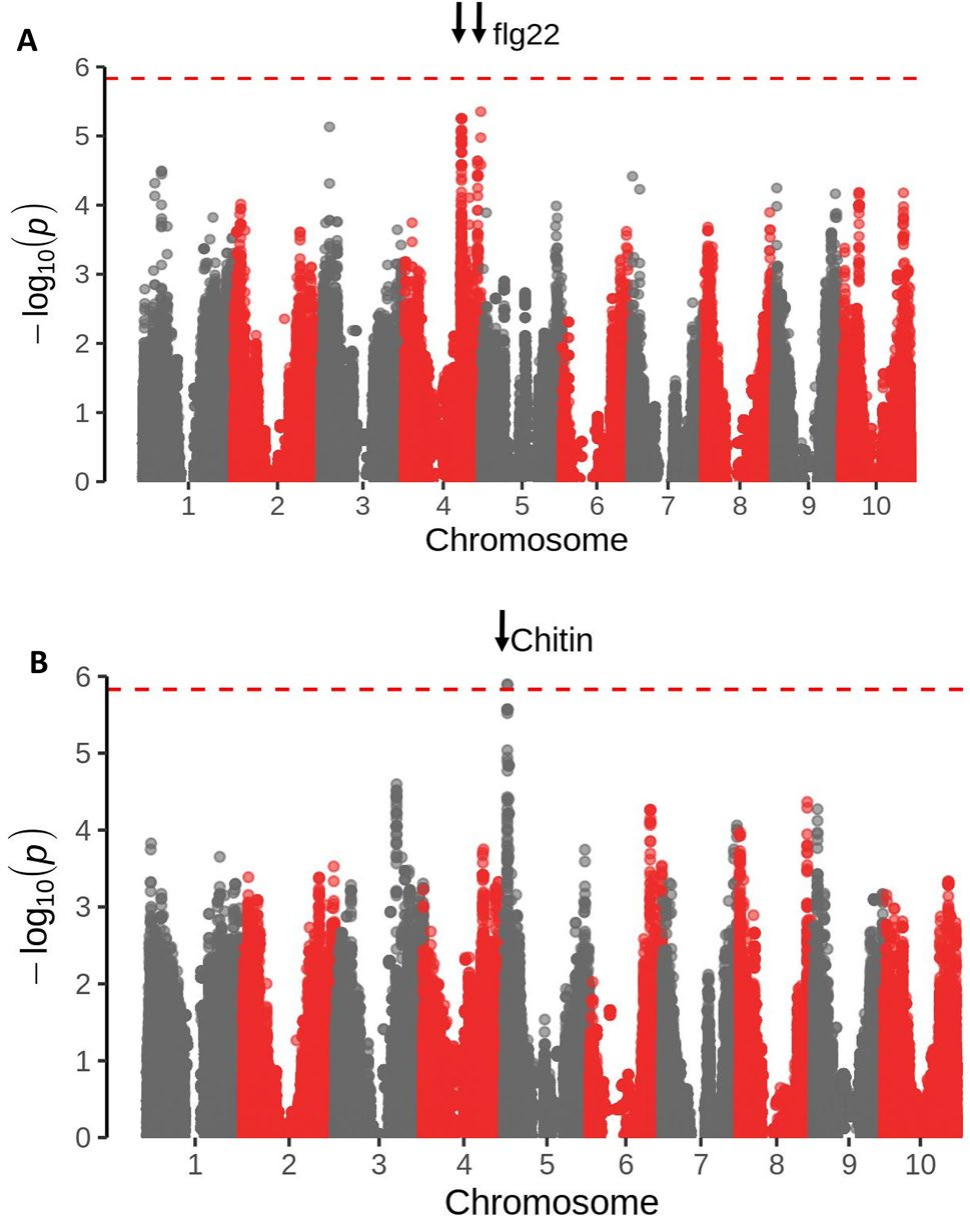
Manhattan plot of GWAS of flg22 (**A**) and chitin (**B**) induced ROS production in SCP X-axis indicates position in the genome. Different chromosomes are illustrated as alternating grey and red bands; Y-axis indicates distribution of –log_10_(p). The Bonferroni multiple comparison test correction significance threshold is indicated. Highly associated SNPs are indicated by arrows.

The phenotypic data were transformed as described and used for association analysis. Q-Q plots did not indicate an excess of false positives (Fig. S3). Two associations with the flg22 response were detected on chromosome 4. The significance threshold was calculated using a Bonferroni multiple comparison test correction which is based on the number of markers used. Since we used a relatively high number of markers (more than 750,000) this threshold was consequently relatively conservative. It should be noted that the associations with flg22 were below the threshold for significance we used but we are nevertheless reporting them as they are the highest associations detected and, given the conservative significance threshold used, are nevertheless likely to reflect real associations. One significantly associated locus was detected on chromosome 5 for the chitin response (Fig. 3). Table 3 shows the parameters associated with these associated loci and details predicted genes. While it is premature to assign causation, it is interesting to note that several of the candidate genes associated with flg22- and chitin-induced responses have homology to genes involved in the defense response or disease resistance in other systems. For instance, the durable wheat rust resistance gene LR34 is an ABC transporter^36^ while genes involved in the auxin response^37^ and the ubiquitin-mediated protein-degradation^38^ pathway have been implicated in disease resistance in other systems.

### Comparison of TLS resistance and MAMP response data

To understand whether variation in the response elicited by flg22 and chitin is connected to TLS resistance, we looked for correlation between MAMP response and TLS disease scores. Despite chitin being an integral component of the fungal cell wall and TLS being a fungal disease, we did not observe a significant correlation between the traits. We did observe a small but moderately-significant negative correlation between the flg22 response and TLS scores (− 0.13*, *p* value < 0.05), indicating that a higher flg22 response was somewhat associated with higher susceptibility. This was unexpected both because flg22 is a bacterial MAMP and TLS is a fungal disease and because we were expecting an association between increased MAMP response and increased resistance. Instead, we observed an opposite relationship, albeit quite weak. Two possible explanations occur to us. Since the correlation is relatively low, this may not be a meaningful correlation. Alternatively, several necrotrophic pathogens of a similar type to *Bipolaris cookei* have effectors that both induce ETI and facilitate pathogenesis. It appears that in these cases elicitation of HR allows the pathogen to grow on the resulting dead host cells^39^. It is possible that this correlation is due to a similar subversion of the plant defense machinery.

A recent companion study measuring the flg22 response and TLS resistance in two sorghum recombinant inbred line (RIL) populations did not identify correlations between the 2 traits or any colocalizing QTL^7^. In the current study, we used the SCP which provided higher genetic and phenotypic diversity than had been available from the two RIL populations but, overall this study also did not produce evidence to support our original hypothesis that a stronger MAMP response is predictive of stronger QDR. However, there are a number of caveats that make it impossible to draw general conclusions.

Perhaps the major caveat is that quantitation of the MTI response is complex. It depends on what MAMP is used and how the response is measured. Low correlations between responses to different MAMPs have been reported previously^19,21,24^, although, as mentioned above, Zhang et al^22^ observed a significant correlation between flg22 and chitin responses in maize. Moreover, the MAMP response can be quantified in a number of different ways, including measurement of ROS or NO production, MAP kinase phosphorylation, mRNA accumulation levels, lignin and cell wall-bound phenols, callose deposition, seedling growth inhibition and MAMP-induced pathogen resistance^18,19,40^. Relative line rankings vary significantly depending on the assay used^19,22^. Our preliminary data also suggests that the MAMP response varies with the age of the plant and the individual leaf on the plant. Essentially, quantification of the MAMP response is complex and inferences may vary significantly depending on how the response is elicited and how measured^7^.

The other major caveat is of course resistance to only one disease was assessed. It may be that resistance to certain diseases, perhaps those that are less well adapted to the host and cannot completely suppress basal resistance mechanisms, may be more associated with the MAMP response. As more diseases are assessed on the SCP we may be able to re-evaluate our hypothesis in the light of multiple comparisons.

## Supplementary information

Supplementary Fig S1.

Supplementary Fig S2.

Supplementary Fig S3.

Supplementary Information.

Supplementary Legends.
