# Supplementary figures and images for: Genome-wide association analysis of the strength of the MAMP-elicited defense response and resistance to target leaf spot in sorghum
Source: Sci Rep. 2020 Nov 30;10:20817. doi: 10.1038/s41598-020-77684-w (PMC7704633; doi:10.1038/s41598-020-77684-w)

## Slide 1
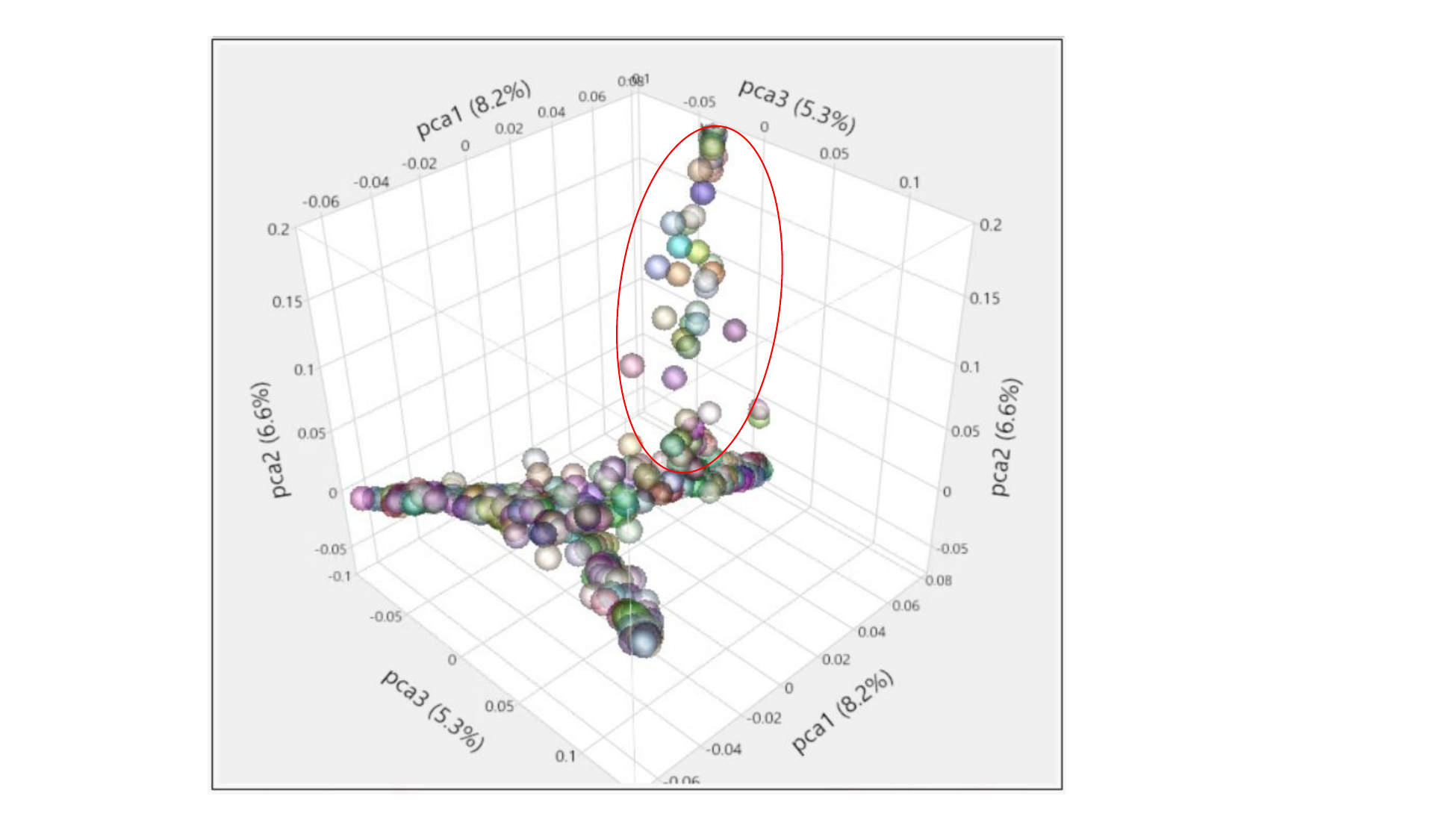

Supplement: Supplementary file 1 — Supplementary Fig S1. [file 41598_2020_77684_MOESM1_ESM.pptx]

## Slide 1
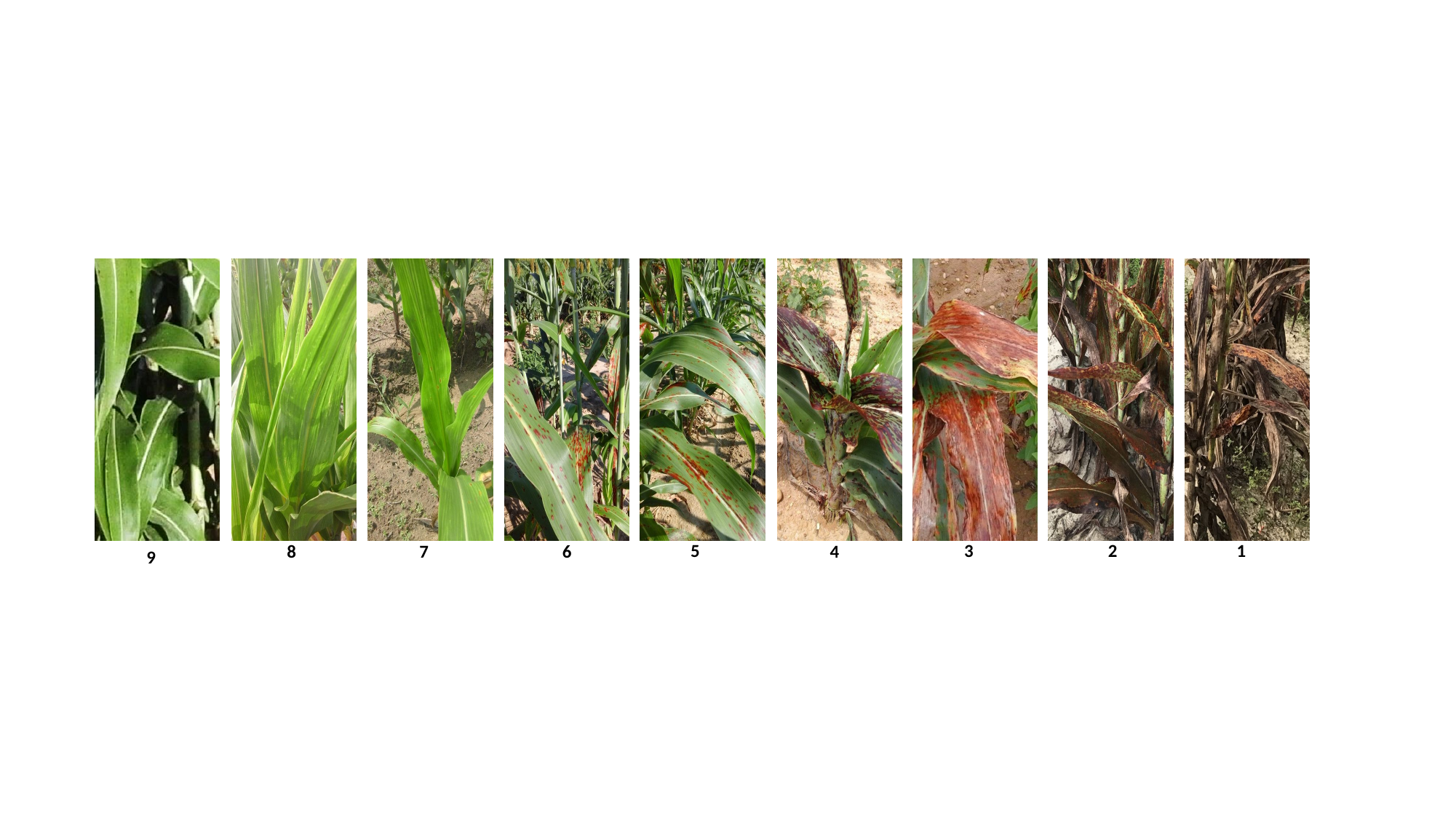

5
2
1
3
8
7
6
4
9

Supplement: Supplementary file 2 — Supplementary Fig S2. [file 41598_2020_77684_MOESM2_ESM.pptx]

## Slide 1
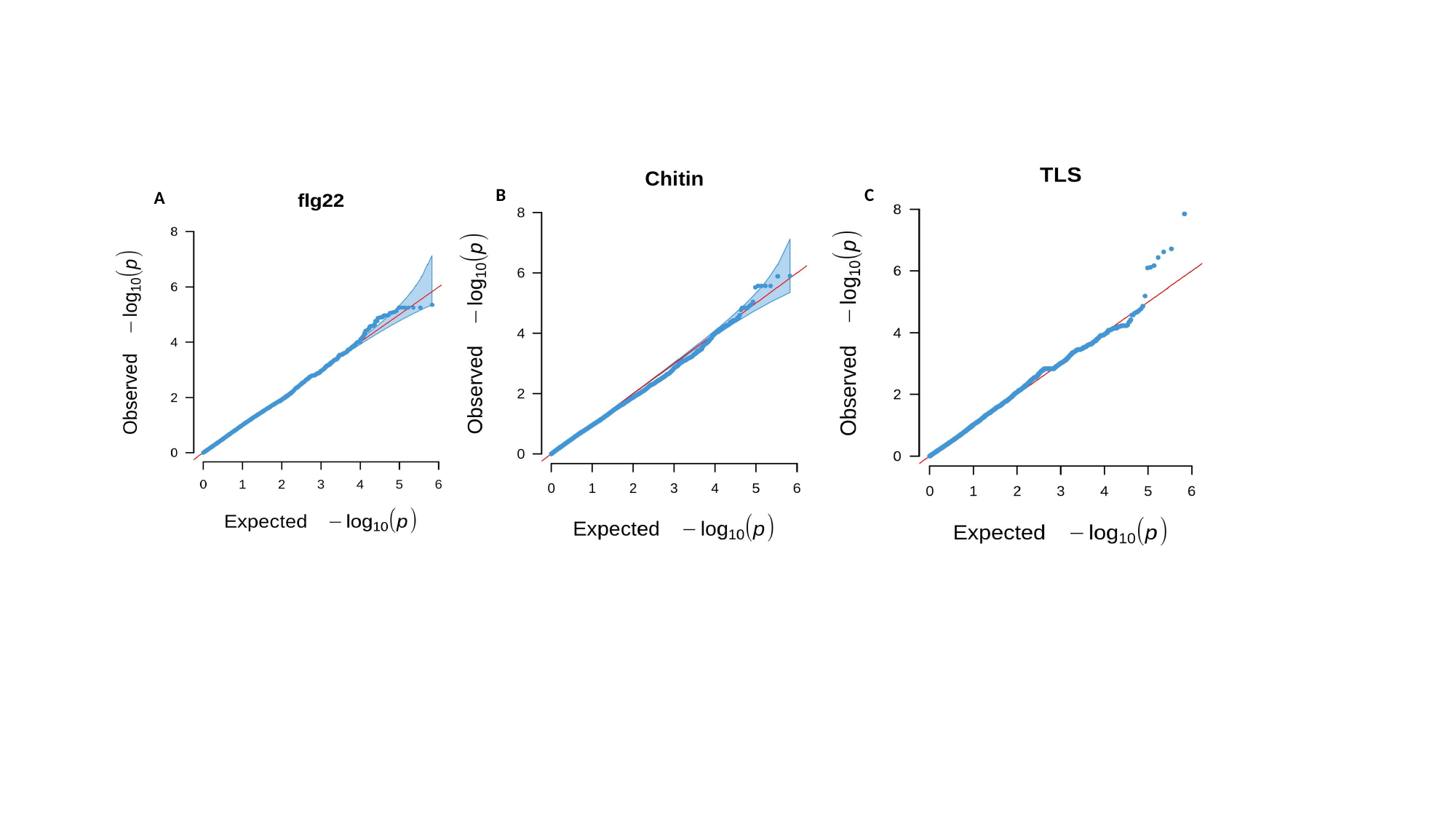

C
B
A

Supplement: Supplementary file 3 — Supplementary Fig S3. [file 41598_2020_77684_MOESM3_ESM.pptx]
