# Supplementary material for: Genome-wide association analysis of the strength of the MAMP-elicited defense response and resistance to target leaf spot in sorghum
Source: Sci Rep. 2020 Nov 30;10:20817. doi: 10.1038/s41598-020-77684-w (PMC7704633; doi:10.1038/s41598-020-77684-w)
Supplement: Supplementary file 5 — Supplementary Legends. [file 41598_2020_77684_MOESM5_ESM.docx]

Supplemental Information for

**Genome-wide Association Analysis of the Strength of the MAMP-elicited Defense Response and Resistance to Target Leaf Spot in Sorghum.**

**Rozalynne Samira^1,8*^, Jennifer A. Kimball^2*†^, Luis Fernando Samayoa Lopez ^3^, James Holland ^3,7^ Tiffany M. Jamann ^4^, Patrick J. Brown^5^, Gary Stacey^6,^ and Peter J. Balint-Kurti^1,7†^**

^1^ Department of Entomology and Plant Pathology, North Carolina State University, Raleigh, NC 27695-

7613, USA

^2^ Department of Agronomy and Plant Genetics, University of Minnesota, 1991 Upper Buford Circle, St.

Paul, MN 55108

^3^ Department of Crop and Soil Sciences, North Carolina State University, Raleigh, NC 27695-7620, USA

^4^ Department of Crop Sciences, University of Illinois,1102 S. Goodwin Ave,Urbana, IL 61801

^5^ Department of Plant Sciences, UC Davis, One Shields Ave, Davis CA 95616

^6^ Divisions of Plant Science and Biochemistry, University of Missouri, Columbia, MO 65211

^7^ USDA-ARS Plant Science Research Unit, Raleigh, NC 27695, USA

^8^ Department of Plant and Soil Science, Texas Tech University, Lubbock, Texas

* These authors should be considered joint first authors

**^†^**Authors for correspondence

PBK [Peter.Balint-Kurti@USDA.GOV](mailto:Peter.Balint-Kurti@USDA.GOV)

JAK jkimball@umn.edu

**Supplementary Figure 1.** Sorghum association panel population: PCA calculated using ~58 Kb SNPs (pairwise R2 > 0.5 were pruned out). The spheres represent each of the inbred lines in the SCP. The spheres in the red oval are outliers that explain more than 7% of the variability. These were excluded from the analysis**.**

**Supplementary Figure 2:** Examples of sorghum lines with different levels of TLS resistance with the allotted score on a 1-9 scale shown beneath each picture.

**Supplementary Figure 3** **:** QQ-Plot of (A) flg22 response, (B) Chitin response and (C) TLS resistance in the SCP.

**Supplemental Files:**

**File S1.** The phenotypic data used in this study.
